# ^1^H NMR Based Metabolomics in Human Sepsis and Healthy Serum

**DOI:** 10.3390/metabo10020070

**Published:** 2020-02-15

**Authors:** Henna Jaurila, Vesa Koivukangas, Marjo Koskela, Fiia Gäddnäs, Sami Myllymaa, Arja Kullaa, Tuula Salo, Tero I. Ala-Kokko

**Affiliations:** 1 Research Group of Surgery, Anesthesia and Intensive Care, Oulu University Hospital, PO. Box 21, 90029 Oulu, Finland; 2 Medical Research Center Oulu, University of Oulu, P.O. Box 5281, 90014 Oulu, Finland; 3 Cancer and Translational Medicine Research Unit, Faculty of Medicine, P.O. Box 5281, University of Oulu, 90014 Oulu, Finland; 4 Department of Applied Physics & SIB Labs, University of Eastern Finland, P.O. Box 1627, 70211 Kuopio, Finland; 5 Institute of Dentistry, Faculty of Health Sciences, University of Eastern Finland, P.O. Box 1627, 70211 Kuopio, Finland; 6 Department of Oral Diagnostics, Educational Dental Clinic, Kuopio University Hospital, P.O. Box 1627, 70211 Kuopio, Finland; 7 Research Group of Oral Health Sciences, Oulu University Hospital, PO. Box 5000, University of Oulu, 90014 Oulu, Finland

**Keywords:** sepsis, human serum, NMR, metabolomics, 3-hydroxybutyrate, citrate, glycine, histidine, AGP

## Abstract

Early diagnosis is essential but challenging in severe sepsis. Quantifying and comparing metabolite concentrations in serum has been suggested as a new diagnostic tool. Here we used proton nuclear magnetic resonance spectroscopy (^1^H NMR) based metabolomics to analyze the possible differences in metabolite concentrations between sera taken from septic patients and healthy controls, as well as between sera of surviving and non-surviving sepsis patients. We took serum samples from 44 sepsis patients when the first sepsis induced organ dysfunction was found. Serum samples were also collected from 14 age and gender matched healthy controls. The samples were analyzed by quantitative ^1^H NMR spectroscopy for non-lipid metabolites. We found that the serum levels of glucose, glycine, 3-hydroxybutyrate, creatinine and glycoprotein acetyls (mostly alpha-1-acid glycoprotein, AGP) were significantly (*p* < 0.05) higher in sepsis compared to healthy sera, whereas citrate and histidine were significantly (*p* < 0.05) lower in sepsis patients compared to healthy controls. We found statistically significantly higher serum lactate and citrate concentrations in non-survivors compared to 30-day survivors. According to our study, 3-hydroxybutyrate, citrate, glycine, histidine, and AGP are candidates for further studies to enable identification of phenotype association in the early stages of sepsis.

## 1. Introduction

Sepsis is defined as a dysregulated host response to pathogens or injury leading to acute organ dysfunction with high morbidity and mortality [1,2]. Early diagnosis and accurate estimation of the disease severity are crucial but challenging due to the heterogeneous manifestations of sepsis. Metabolic dysfunction is thought to be a key factor in the development and response to sepsis [3]. Biomarkers analyzed from blood samples, such as procalcitonin (PCT) and lactate, are regularly used in sepsis evaluation in intensive care units (ICU). These biomarkers are thought to show the presence of infection and disease severity [4]. Repeated measurements of biomarkers can also help to evaluate the clinical course and prognosis [5,6]. However, the sensitivity and specificity of a single biomarker are not sufficient due to the complex pathogenesis of sepsis [4,7].

Metabolomics (or metabonomics) is a rapidly emerging discipline that aims to identify and quantify the changes in concentrations of the metabolites in a biological sample, such as blood, urine, or saliva. Metabolites are chemical fingerprints that cellular processes leave behind. Nuclear magnetic resonance (NMR) spectroscopy is a quantitative high throughput method that is non-destructive for the sample and needs only minimal sample preparation. NMR spectra and metabolites in human serum have been identified earlier [8] and some have combined multivariate statistical analysis and pattern recognition with NMR data to distinguish critically ill patients from healthy controls [7,9,10]. However, the few studies in which human sepsis metabolites in blood are analyzed with NMR spectroscopy report conflicting results [7,10,11,12,13,14,15,16].

In this study, we hypothesized that there are differences in metabolite concentrations in proton nuclear magnetic resonance (^1^H NMR) spectroscopy analysis between sera taken from septic patients and healthy controls. These alterations in concentrations could describe metabolic derangements in sepsis and their association to the severity of illness and mortality. We explored whether there are any definitive metabolic changes between surviving and non-surviving patient sera. We also compared our results to those obtained in earlier studies to determine the possible similarities and differences between the various studies. We aimed to find candidates for sepsis-related metabolites to study further their diagnostic or prognostic purposes.

## 2. Results

### 2.1. Patients

This study is a secondary analysis of a previously published cohort [17]. The key characteristics of the patient population are shown in Table 1. The majority of the 44 patients were male (66%), and there were 33 survivors (75%) on day 30. The control group consisted of 14 healthy age and gender matched volunteers, eight of them men (57%). The median age of the control group was 61 years (25th to 75th percentile, 56 to 69 years).

### 2.2. Serum Metabolites

We compared the concentrations of metabolites in the sera collected from the sepsis patients (*n* = 44) and the healthy controls (*n* = 14). We focused on the potentially relevant non-lipid metabolites which have been associated to sepsis in previous human blood NMR studies [7,10,11,12,13,14,15,16]. We identified and quantified 20 non-lipid metabolites and compared the metabolite concentrations between sepsis and control groups (Table 2), as well as between sepsis survivor and non-survivor groups (Table 3). Some metabolites could not be analyzed reliably enough due to sample irregularities (mainly related to low protein content, polysaccharides, and abnormal macromolecule A) or they were rejected by the automatic sample and measurement quality control (Table S1). Thus, the concentrations of pyruvate, glycerol, glutamine, leucine, phenylalanine, acetate, and acetoacetate were disqualified. In survivor and non-survivor analyses creatinine also was excluded. Furthermore, albumin was rejected due to known uncertainties in changing spectral signal areas to albumin concentrations.

Between sepsis patients and controls, we found significant differences in the serum concentrations of the following eight metabolites: glucose (median concentration 5.63 mmol/l vs 4.13 mmol/l, *p* < 0.01), citrate (0.08 mmol/l vs 0.10 mmol/l, *p* < 0.01), glycine (0.43 mmol/l vs 0.31 mmol/l, *p* < 0.001), histidine (0.07 mmol/l vs 0.08 mmol/l, *p* < 0.05), 3-hydroxybutyrate (0.15 mmol/l vs 0.11 mmol/l, *p* < 0.01), creatinine (0.11 mmol/l vs 0.05 mmol/l, *p* < 0.001), and glycoprotein acetyls (mainly alpha−1-acid glycoprotein, AGP) (1.81 mmol/l vs 1.34 mmol/l, *p* < 0.001) (Table 2). We did not find any significant differences in concentrations of lactate, alanine, isoleucine, valine, or tyrosine between sepsis and healthy sera. Between sepsis survivors and non-survivors, we found significant differences only in the lactate (median concentration 1.46 mmol/l vs 2.49 mmol/l, *p* < 0.01) and citrate concentrations (0.08 mmol/l vs 0.10 mmol/l, *p* < 0.05) (Table 3).

We calculated correlations between statistically significant metabolites (glucose, citrate, glycine, histidine, 3-hydroxybutyrate, creatinine, glycoprotein acetyls) and clinical variables (30-day mortality, septic shock, APACHE II score on admission, SOFA score on admission, maximum SOFA score during the ICU period, length of stay at the ICU). The significant (*p* < 0.05) correlations (*r*) are presented in Table 4. There were no significant correlations in metabolites and septic shock, APACHE II score or length of stay (not shown).

## 3. Discussion

We analyzed sera from adult sepsis patients and matched healthy controls by using ^1^H NMR spectroscopy. We found that the levels of glucose, glycine, 3-hydroxybutyrate, creatinine and glycoprotein acetyls (mostly alpha-1-acid glycoprotein, AGP) were significantly increased in sepsis compared to healthy sera, whereas citrate and histidine were significantly decreased in sepsis patients compared to healthy controls. Citrate and histidine correlated with the severity of organ dysfunction. When comparing sepsis survivors and non-survivors, we found a significant increase only in the serum lactate and citrate concentrations. Our study had a control group of age and gender matched healthy individuals. The majority of the earlier studies used either non-sepsis ICU patients, emergency room sepsis, or trauma patients as controls [12,13,14,15], which complicates the comparison between studies.

There are a few earlier human studies using NMR spectroscopy to identify and quantify metabolites from sepsis sera or plasma [7,10,11,12,13,14,15,16]. Similar to our study two of them compared samples taken from adult sepsis patients and healthy controls [7,11], and one study compared analogous samples from a pediatric population [10] (Table 5). Two of these focus on pattern recognition and multivariate statistical analysis [7,10], and one has an aberrant reporting method of the results [11]. In a study comparing metabolic changes in plasma connected to sepsis-induced acute lung injury (ALI) [11], the findings diverged from ours or other similar studies (Table 5). This may be due to the differences in reporting their results using false discovery rate (FDR) combined with Student’s *t*-test [11].

We found that glucose was upregulated in sepsis patients, which is in line with a previous pediatric sepsis study [10]. Hyperglycemia, combined with insulin resistance, is a common and multifactorial adaptive response in sepsis [18]. In the study of Singh, they reported depletion of blood glucose in sepsis cases compared to healthy controls. However, this was seen in a representative ^1^H NMR spectra of one patient while significantly elevated mean value of glucose was demonstrated in the sepsis group in their supplemental data similar to our results.

We found an increased concentration of 3-hydroxybutyrate in sepsis sera compared to control sera, which is also in line with the findings of the pediatric sepsis study [10]. Increased levels of ketone bodies, such as hydroxybutyrate, acetoacetate, and acetone, are signs of fatty acid breakdown. This is a marker of the increased energy requirement during infection [10,19]. Blaise et al. investigated trauma patients in order to find the metabolic phenotype susceptible to sepsis development [12]. They found, as we did, that 3-hydroxybutyrate correlated with the later development of sepsis. Increased concentrations of 3-hydroxybutyrate have also been detected in two adult sepsis studies comparing sepsis patients to ICU controls [13,14] and a pediatric study comparing ICU sepsis patients to emergency department (ED) sepsis patients [15]. In a recent study, septic non-survivors had elevated levels of 3-hydroxybutyrate in serum compared to survivors [16]. Our finding confirms that in sepsis ketone levels increase.

We observed varying differences in amino acid serum levels between sepsis patients and healthy controls. Citrate is a key regulator in energy production and an essential intermediate in metabolism. Citrate inhibits the adenosine triphosphate (ATP) producing processes of glycolysis, fatty acid beta-oxidation, and tricarboxylic acid (TCA) cycle as well as stimulates ATP consuming gluconeogenesis and lipid synthesis. In inflammation, citrate is reduced to its derivatives in the cytosol to supply components to altered energy and intermediate production [20]. Citrate contributes to the production of prostaglandin, reactive oxygen species (ROS), and nitric oxide (NO), which have a remarkable role in inflammation and cell damage [20]. In normal conditions plasma citrate is mostly extracted via the kidneys, but under surgical stress the hepatic uptake increases and leads to hypocitricemia [21]. Thus, both uptake into the cells and upregulated extraction might explain the decreased citrate levels in sepsis serum in our study. In the pediatric study of Mickiewicz et al. [10], citrate was also found to be decreased. In contrast to these results, increased citrate concentrations have been found in the sera of urosepsis patients in comparison to that of healthy controls [7]. The renal clearance of citrate is essential for the removal of citrate from plasma and the body [21] and thus might decline in urological patients. In the study of trauma patients, citrate concentrations correlated with later development of sepsis [12]. We also found increased glycine levels in sepsis. Altered levels of glycine are connected to amino acid oxidation [22]. In a rodent sepsis model in which metabolic markers in rat liver tissue were examined, an increased TCA cycle with peripheral release of amino acids like glycine for the liver metabolism and an increase in TCA intermediate products including citrate were reported [23]. In our series, histidine levels were lower in sepsis than in controls. In contrast, in the pediatric study, histidine levels in sepsis were increased [10]. Histidine has anti-inflammatory and anti-oxidant properties [24], and low plasma histidine concentrations are associated with oxidative stress, inflammation, protein-energy wasting, and mortality in patients with chronic kidney disease [25]. This kind of increased consumption of amino acids may also explain the lower than healthy levels in our series and the different finding in the pediatric study. Decreased amino acid concentration in blood and yet enhanced hepatic uptake and augmentation of hepatic extraction of amino acids in sepsis has also been confirmed by others [26]. Taken together, enhanced peripheral protein catabolism could explain the increased level of glycine in sepsis sera while concomitant stimulation of hepatic extraction and metabolism may explain the lower levels of histidine.

Identical to our results, in the pediatric sepsis study by Mickiewicz et al. [10], there was a significantly increased concentration of creatinine in sepsis sera compared to the healthy controls’ sera. Increased creatinine is construed as a sign of kidney failure [27,28].

In our study, glycoprotein acetyls consisted mostly of alpha-1-acid glycoprotein (AGP, also known as orosomucoid). To our knowledge, no previous NMR based analysis of AGP levels in sepsis has been reported. AGP has many functions: it modulates immunity, binds and transports drugs and maintains capillary barrier function [29,30]. AGP is an acute phase protein, and it is elevated in response to infection and inflammation [31]. Serum levels of AGP have been found to be higher in the sepsis group compared to the systemic inflammatory response syndrome (SIRS) group, and it is speculated that it could be used to assess the severity of sepsis [32]. Furthermore, AGP and citrate have been identified in an NMR study as two of four predictive biomarkers in blood to estimate the short-term risk of death from any cause in two general population cohorts, AGP being the strongest biomarker [33]. Our finding of an increased level in sepsis also supports the idea of its value in the prediction of sepsis.

In our series, non-survivors had significantly increased lactate and citrate levels compared to survivors, and citrate levels correlated positively with the level of organ dysfunctions, which is supported by the recent study of Liu et al. [16]. In their study, metabolic dysregulation and increase in amino acids due to significant protein breakdown were associated with a poor outcome in septic shock. Imbalance between oxygen delivery and consumption leads to anaerobic metabolism and lactate production [34]. The pediatric study was the only one to find altered lactate levels in sepsis compared to healthy controls [10]. It has been suggested that early lactate clearance is associated with better outcomes in sepsis [34], which is supported by our findings. The increased citrate level could be explained by severe metabolic disorder and organ dysfunctions. Recently, there have been studies exploring targeted metabolomics profiles in sepsis, focusing on metabolic changes associated with mortality with pattern recognition [10,13,14,16,35,36]. The only common result for these studies seems to be that non-survivors tend to have extensive metabolic disorders, and most metabolite levels are higher than in survivors. Mickiewicz et al. and Liu et al. were able to distinguish non-survivors from survivors accurately with a predictive NMR model [10,13,14,16].

This was a single center observational study with a relatively small sample size. The small sample size prevented us from using statistical modeling such as principal component analysis (PCA) to generate a model of prediction using these biomarkers as an early diagnostic tool during sepsis. Consequently, the results should be interpreted as hypothesis generating. In all sepsis studies there is a difficulty in timing the initiation of this heterogenous disease, which makes the comparison of samples from different patients challenging, especially in metabolomics. To control the quality of our study samples, we compared them to clinical blood samples taken according to the normal ICU protocol during the same day. The controls were healthy individuals leaving the possibility that the differences detected are not necessarily specific for infection but reflect disease severity and organ dysfunctions. Comparing sepsis patients with healthy controls is an academic approach and does not resemble the diagnostic dilemma. Future studies should include other critically ill patients without underlying infection, as well as critically ill patients (e.g., systemic inflammatory response syndrome or quickSOFA positive) with suspected infection. These patients could be assessed and grouped into infection and non-infection post-hoc. It would also be interesting to study the impact of different infectious origins on the metabolic profile.

Taken together, based on our results and the current literature, the altered levels of metabolites in the blood of sepsis patients are mostly related to energy metabolism, oxidative stress and organ dysfunction. There is no single biomarker but rather several biomarkers, which should be taken as hints of the pathological mechanisms involved. It was shown in an animal study that changes in the serum metabolic profile were earlier than those of organ dysfunction [37]. Thus, metabolomics is a promising technique that could help to determine the timely diagnosis of sepsis. In the future, measuring multiple metabolites at different time points in sepsis will hopefully help us to understand the pathophysiology of the host response. In that way, we might learn what part of the host response is adaptive and what are the alterations that lead to irreversible organ failure and death. According to our study, 3-hydroxybuturate, citrate, glycine, histidine and AGP could be the candidates for further studies.

## 4. Material and Methods

### 4.1. Patients

The study was conducted in a 12-bed mixed, adult intensive care unit in Oulu University Hospital, Oulu, Finland. Of the 1361 patients admitted to the ICU at Oulu University Hospital during the 20 months study period, 238 had severe sepsis and 66 met the inclusion criteria. The inclusion criteria for this study were diagnosis of severe sepsis according to the American College of Chest Physicians/Society of Critical Care Medicine criteria [1]. The exclusion criteria were: age under 18 years, surgery not related to sepsis, surgery during the preceding 6 months, malignancy, bleeding disorder, and chronic renal or hepatic failure and immunosuppressive treatment not related to sepsis. Patients entered the study when a diagnosis of severe sepsis was set and within 24 h after the first organ dysfunction was found. The patients were treated according to the normal ICU protocol and severe sepsis guidelines [38]. Normoglycemia was targeted with insulin infusion. The study was approved by the local ethics committee. Written informed consent was obtained from 44 patients or their next of kin. This is a continuation of an earlier study looking at skin wound healing in severe sepsis [17].

The following information was collected from the patients: age, gender, chronic diseases, type of ICU admission (medical or surgical), reason for admission, focus of infection, prevalence of septic shock, severity of underlying diseases on admission as assessed by APACHE II, and evolution of daily organ dysfunctions assessed by daily SOFA scores. The length of stay at the ICU was recorded as well as 30-day mortalities. Fourteen healthy gender and age matched volunteers served as controls.

### 4.2. Blood Samples

Serum samples were collected within 24 h from the detection of the first sepsis induced organ dysfunction. SOFA score 1 to 2 in one organ system on one or more days during the study period was defined as organ dysfunction [1,39]. Serum samples from the healthy controls were taken as well. Blood was collected into 10 mL plain red cap vacutainer glass tubes without any additional ingredients. The handling process was performed by our accredited central laboratory within 2 h in stable conditions [40]. After 30 min of clotting in room temperature samples were centrifuged (2000× *g* for 10 min in 20 °C), separated, and frozen first in −25 °C and stored in −70 °C within 24 h. As a quality control, we compared the metabolite concentrations of fresh clinical samples with the study samples of these patients, and we detected that glucose, lactate, and creatinine values were within range. In the clinical samples, the mean value of the minimum glucose values during the study day was 5.4 mmol/L, and the mean value of the maximum glucose values was 10.4 mmol/L, while the mean value of the study samples was 5.7 mmol/L. The mean value of the minimum clinical lactate values was 2.27 mmol/L, and the mean value of maximum values was 3.4 mmol/L, while the mean value of the study samples was 2.26 mmol/L. The mean value of creatinine was 0.143 mmol/L in the clinical samples while that of the study samples was 0.126 mmol/L.

### 4.3. Proton Nuclear Magnetic Resonance Spectroscopy

Proton nuclear magnetic resonance (^1^H NMR) spectroscopy exploits the magnetic properties of the atomic nuclei in a molecule and the changes in the resonance frequency of the nuclei [41]. Protons resonate in a strong magnetic field, and every metabolite has its own unique spectrum that represents the environment of each proton. In our study, 260 µL of serum and 260 µL of sodium phosphate buffer (75 mmol/L Na_2_HPO_4_ in 80%/20% H_2_O/D_2_O, pH 7.4) were carefully mixed and transferred to the NMR tubes. The high-resolution one-dimensional (1D) proton (^1^H) NMR spectrum was acquired using a Bruker AVANCE III HD spectrometer (Bruker, Billerica, MA, USA) operating at 600.20 MHz and equipped with an inverse triple resonance cryoprobe (Bruker CryoProbe Prodigy). The spectrometer was controlled via TopSpin 3.2 (Bruker) software. Before the measurement, the samples were preheated to 37 °C and then automatically shimmed using the TopShim routine (Bruker). The samples were handled in 96-well plates and every plate contained two quality control samples, a serum mimic and a mixture of two low-molecular-weight metabolites. The former was used to monitor the consistency of quantifications, whereas the latter was a technical reference to monitor the performance of the spectrometer [42]. For all the samples, the lipoprotein (LIPO) and low-molecular-weight metabolites (LMWM) data were automatically collected. The LIPO data were collected applying a Bruker noesypresat pulse sequence. The LIPO window is dominated by broad signals arising from macromolecules, mainly lipoprotein lipids and albumin. In the LMWM window a CPMG pulse sequence that suppressed the macromolecule signals was applied, thus enhancing the detection of smaller molecules. After the collection, the samples went through a standardized multi-step lipid extraction procedure, after which the extracted lipid (LIPID) data was collected. A constant receiver gain setting was used for all the samples. The raw NMR spectra were manually corrected for phase using TopSpin 3.0 software (Bruker BioSpin GmbH). The constrained total-line-shape fitting tool in PERCH NMR software (PERCH Solutions Ltd., Kuopio, Finland) [43] was used to quantify the metabolites found. This method enables accurate quantification of the identified metabolites even if the baseline is not linear or signals overlap [43]. The signal areas were referenced to an internal reference compound (trimethylsilylpropanoic acid, TSP). However, it is well known that TSP binds to albumin, and the bound fraction becomes invisible in the NMR spectra [44]. Due to this fact, the use of TSP as an internal standard for low-molecular weight molecules is problematic. However, spectral information of the actual sample also underwent various comparisons with the spectra of two quality control samples. The information achieved by control samples were used to help in a regression model to produce reliable quantified data. A more detailed description of the experimentation has been presented earlier [42,45]. The final metabolite concentrations are reported as mmol/l in serum.

### 4.4. Statistical Analysis

The data were entered into an SPSS database for analysis (SPSS version 24, IBM SPSS Statistics, Chicago, IL, USA). Comparisons between groups were performed using the independent-samples *t*-test or the Mann–Whitney U test depending on the normality of the data. Cross-tabulation was used to describe the relationship between two categorical variables, and the statistical significance was then calculated with Fischer’s exact test. Two-tailed *p* values were reported and differences were considered significant at *p* < 0.05. The correlations between metabolites and clinical variables were calculated using either Pearson’s or Spearman’s method depending on the normality of the data. The *p* values were significant when under 0.05.

### 4.5. Ethics Approval and Consent to Participate

This study was conducted in accordance with the Declaration of Helsinki, and the study protocol was approved by The Regional Ethics Committee of the Northern Ostrobothnia Hospital District. Written informed consent was obtained from each patient or their next of kin.

## Figures and Tables

**Table 1:** Patient demographics. All patients, sepsis 30-day survivors and non-survivors are listed. Data are expressed as medians and 25th to 75th percentiles or with frequencies and percentages. *p* value is statistically significant when <0.05 and those values are marked with an *. ICU = intensive care unit; APACHE II = acute physiology and chronic health evaluation II; SOFA = sequential organ failure assessment; MODS = multiple organ dysfunction syndrome; MOF = multiple organ failure.

Characteristics	Variables			
	All Patients (*n* = 44)	Survivors (*n* = 33)	Non-Survivors (*n* = 11)	*p* Value Survivors vs. Non-Survivors
Male gender, *n* (%)	29 (66%)	20 (61%)	9 (82%)	0.282
Age, years	63 (56 to 71)	61 (55 to 67)	71 (61 to 75)	0.063
Septic shock, *n* (%)	38 (86%)	27 (82%)	11 (100%)	0.311
Length of stay in the ICU, days	7 (4 to 12)	6 (4 to 10)	11 (6 to 16)	0.159
APACHE II on admission, points	26 (22 to 31)	24 (22 to 28)	31 (26 to 38)	<0.01*
SOFA score on admission, points	8 (6 to 12)	8 (6 to 11)	11 (8 to 15)	0.083
SOFA score maximum, points	10 (7 to 16)	8 (7 to 12)	16 (10 to 21)	<0.01*
MODS, *n* (%)	14 (32%)	13 (39%)	1 (9%)	0.076
MOF, *n* (%)	30 (68%)	20 (61%)	10 (91%)	0.076
Focus of infection, *n* (%)				
Lungs	18 (41%)	13 (39%)	5 (46%)	0.738
Intra-abdominal	16 (36%)	12 (36%)	4 (36%)	1.000
Primary blood	3 (7%)	2 (6%)	1 (9%)	1.000
Urinary	1 (2%)	1 (3%)	0	1.000
Other	6 (14%)	5 (15%)	1 (9%)	1.000
Chronic diseases, *n* (%)				
Coronary artery disease	9 (20%)	5 (15%)	4 (36%)	0.195
Chronic obstructive pulmonary disease	5 (11%)	4 (12%)	1 (9%)	1.000
Asthma	4 (9%)	4 (12%)	0 (0%)	0.558
Diabetes	10 (23%)	9 (27%)	1 (9%)	0.408
Arteriosclerosis obliterans	4 (9%)	3 (9%)	1 (9%)	1.000
Hypertension	17 (39%)	13 (39%)	4 (36%)	1.000

**Table 2:** Comparison of metabolite concentrations in sera between healthy controls and sepsis patients. Serum levels are presented as mmol/L. Median concentrations with 25th to 75th percentiles are shown. *p* < 0.05 is considered significant, and those values are marked with an *. bOHBut = 3-hydroxybutyrate.

Metabolite	Healthy	Sepsis	*p*-Value	Cases *n* Sepsis (%) and *n* Control (%)
Glucose	4.13 (3.60 to 4.65)	5.63 (4.65 to 6.74)	<0.01*	44 (100 %) and 14 (100 %)
Lactate	1.44 (1.30 to 2.24)	1.61 (1.23 to 2.70)	0.52	44 (100 %) and 14 (100 %)
Citrate	0.10 (0.10 to 0.12)	0.08 (0.06 to 0.11)	<0.01*	41 (93 %) and 14 (100 %)
Alanine	0.50 (0.45 to 0.57)	0.45 (0.34 to 0.66)	0.533	44 (100 %) and 14 (100 %)
Glycine	0.31 (0.29 to 0.34)	0.43 (0.35 to 0.89)	<0.001*	44 (100 %) and 14 (100 %)
Histidine	0.08 (0.08 to 0.10)	0.07 (0.05 to 0.09)	<0.05*	44 (100 %) and 14 (100 %)
Isoleucine	0.07 (0.06 to 0.09)	0.06 (0.05 to 0.08)	0.247	44 (100 %) and 14 (100 %)
Valine	0.22 (0.18 to 0.25)	0.18 (0.13 to 0.21)	0.1	44 (100 %) and 14 (100 %)
Tyrosine	0.07 (0.06 to 0.08)	0.05 (0.04 to 0.07)	0.089	44 (100 %) and 14 (100 %)
bOHBut	0.11 (0.09 to 0.14)	0.15 (0.12 to 0.21)	<0.01*	44 (100 %) and 14 (100 %)
Creatinine	0.05 (0.05 to 0.06)	0.11 (0.07 to 0.17)	<0.001*	30 (68 %) and 14 (100 %)
Glycoprotein acetyls	1.34 (1.24 to 1.61)	1.81 (1.50 to 2.14)	<0.001*	44 (100 %) and 14 (100 %)

**Table 3:** Comparison of metabolite concentrations in sera between sepsis survivors and non-survivors. Serum levels are presented as mmol/L. Median concentrations with 25th to 75th percentiles are shown. *p* < 0.05 is considered significant, and those values are marked with an *. bOHBut = 3-hydroxybutyrate.

Metabolite	Survivor	Non-Survivor	*p*-Value	Cases *n* Survivor (%) and Non-Survivor (%)
Glucose	5.74 (4.51 to 7.00)	5.44 (4.93 to 6.38)	0.44	33 (100 %) and 11 (100 %)
Lactate	1.46 (1.20 to 2.18)	2.49 (2.01 to 4.07)	<0.01*	33 (100 %) and 11 (100 %)
Citrate	0.08 (0.06 to 0.09)	0.10 (0.08 to 0.16)	<0.05*	31 (94 %) and 10 (91 %)
Alanine	0.50 (0.34 to 0.64)	0.45 (0.27 to 1.32)	0.915	33 (100 %) and 11 (100 %)
Glycine	0.47 (0.35 to 0.88)	0.39 (0.34 to 1.08)	0.894	33 (100 %) and 11 (100 %)
Histidine	0.06 (0.05 to 0.08)	0.08 (0.05 to 0.16)	0.317	33 (100 %) and 11 (100 %)
Isoleucine	0.06 (0.05 to 0.08)	0.07 (0.04 to 0.09)	0.815	33 (100 %) and 11 (100 %)
Valine	0.18 (0.13 to 0.21)	0.18 (0.09 to 0.27)	0.931	33 (100 %) and 11 (100 %)
Tyrosine	0.05 (0.04 to 0.06)	0.07 (0.03 to 0.10)	0.280	33 (100 %) and 11 (100 %)
bOHBut	0.15 (0.12 to 0.20)	0.15 (0.11 to 0.34)	1.000	33 (100 %) and 11 (100 %)
Glycoprotein acetyls	1.73 (1.51 to 2.03)	1.99 (1.46 to 2.45)	0.626	33 (100 %) and 11 (100 %)

**Table 4:** Correlations between metabolites and clinical variables. The *p* values are considered significant when <0.05 and marked with an *. SOFA = sequential organ failure assessment; bOHBut = 3-hydroxybutyrate.

Metabolite	Clinical Variable			Statistical Test
	30-day Mortality	SOFA Score on Admission	SOFA Maximum	
bOHBut	*r* = 0.000 *p* = 1.000	*r* = 0.015 *p* = 0.924	*r* = 0.099 *p* = 0.523	Spearman’s correlation
Citrate	*r* = 0.341 *p* < 0.05*	*r* = 0.366 *p* < 0.05*	*r* = 0.472 *p* < 0.01*	Spearman’s correlation
Creatinine	*r* = 0.076 *p* = 0.694	*r* = 0.659 *p* < 0.001*	*r* = 0.636 *p* < 0.001*	Spearman’s correlation
Glucose	*r* = −0.090 *p* = 0.560	r= −0.193 *p* = 0.211	r= −0.176 *p* = 0.253	Pearson’s correlation
Glycine	*r* = −0.023 *p* = 0.884	*r* = 0.151 *p* = 0.327	*r* = 0.254 *p* = 0.096	Spearman’s correlation
Glycoprotein acetyls	*r* = 0.116 *p* = 0.454	r= −0.089 *p* = 0.566	*r* = 0.088 *p* = 0.569	Pearson’s correlation
Histidine	*r* = 0.161 *p* = 0.296	*r* = 0.299 *p* < 0.05*	*r* = 0.362 *p* < 0.05*	Spearman’s correlation

**Table 5:** Studies comparing blood samples between patients with sepsis and healthy controls analyzed with proton nuclear magnetic resonance (1H NMR) spectroscopy. The metabolites are categorized as concentrations higher or lower in sepsis compared to healthy controls. We chose metabolites that were statistically significant in two-samples *t* tests or in PCA/(O)PLS-DA/DFA models, and we show only those metabolites presented in more than one of the table studies. ALI = acute lung injury; ARDS = acute respiratory distress syndrome; FDR = false discovery rate; (P)ICU = (pediatric) intensive care unit; SIRS = systemic inflammatory response syndrome; PCA = principal component analysis; (O)PLS-DA = (orthogonal) partial least square discrimination analysis; (AU)ROC = (area under) receiver operator curve; PCT = procalcitonin; DFA = discriminant function analysis, bOHBut = 3-hydroxybutyrate.

Title	Age Group		Sample	Number of Participants		Patient Population				Computational Analysis Method			
**Stringer et al.** **Am J Physiol Lung Cell Mol Physiol 2011 [11]**	Adult (>18 y)		Plasma, taken in the morning	13 sepsis patients and 6 healthy controls		<7 days from the onset of Sepsis-induced ALI/ARDS				Comparison of concentrations with Student’s t-test (*p* ≤ 0.05) and FDR (*q*- value ≤ 0.05), Spearman’s rank correlation coefficient			
**Mickiewicz et al. Am J Resp Crit Care Med 2013 [10]**	Pediatric (<11 y)		Serum, <24 h of diagnosis of septic shock	60 septic shock patients and 40 healthy controls, also 40 PICU patients with SIRS		Diagnosis of septic shock at the PICU				PCA, PLS-DA, OPLS-DA to separate metabolic variation; AUROC for OPLS-DA			
**Singh et al.** **Clin Chim Acta 2016 [7]**	Adult (>18 y)		Serum	35 urosepsis patients and 32 healthy controls		Urosepsis cases were identified by measuring serum PCT levels (>0.5 ng/mL)				PCA, PLS-DA to separate metabolic variation; ROC for PLS-DA; independent t-test followed by DFA to identify significant biomarkers (*p* < 0.05)			
**Current study**	Adult (>18 y)		Serum, <24 h of the diagnosis of severe sepsis	44 sepsis patients and 14 healthy controls		Admission after the identification of the first sepsis-related organ dysfunction at the ICU				Comparison of concentrations with Student’s t-test or Mann-Whitney test (*p* < 0.05), Pearson’s or Spearman’s correlation coefficients			
	Higher concentrations in sepsis										Lower concentrations in sepsis		
	bOHBut	Acetate		Acetone	Citrate	Creatinine	α-Glucose	Glucose	Histidine	Phenylalanine	Acetate	Citrate	Histidine
Stringer et al. Am J Physiol Lung Cell Mol Physiol 2011 [11]	-	-		-	-	-	-	-	-	-	-	-	-
Mickiewicz et al. Am J Resp Crit Care Med 2013 [10]	×	-		×	-	×		×	×	×	×	×	-
Singh et al. Clin Chim Acta 2016 [7]	-	×		×	×	-	×	-	-	×	-	-	-
Current study	×	-		-	-	×	-	×	-	-	-	×	×

## Supplementary Materials

The following are available online at https://www.mdpi.com/2218-1989/10/2/70/s1. Table S1: Case processing summary.
