# Supplementary material for: 1H NMR Based Metabolomics in Human Sepsis and Healthy Serum
Source: Metabolites. 2020 Feb 15;10(2):70. doi: 10.3390/metabo10020070 (PMC7074315; doi:10.3390/metabo10020070)
Supplement: Supplementary file 1 [file metabolites-10-00070-s001.pdf]

Table S1. Case processing summary. NA: value was rejected by automatic sample and measurement quality control; TAG: value cannot be quantified due to detected irregularity in sample. Rejected metabolites are marked in red font. Please note that albumin was rejected due to the quantification uncertainties.

		**Valid cases**	**Missing cases**	**TAG**	**NA**
**Metabolite**	**Group**	**N**	**N**	**N**	**N**
Glucose	Patients	44	0	0	0
	Controls	14	0	0	0
Lactate	Patients	44	0	0	0
	Controls	14	0	0	0
Pyruvate	Patients	6	38	36	2
	Controls	10	4	0	4
Citrate	Patients	41	3	3	0
	Controls	44	0	0	0
Clycerol	Patients	17	27	9	18
	Controls	6	8	8	0
Alanine	Patients	44	0	0	0
	Controls	14	0	0	0
Glutamine	Patients	8	36	36	0
	Controls	14	0	0	0
Glysine	Patients	44	0	0	0
	Controls	14	0	0	0
Histadine	Patients	44	0	0	0
	Controls	14	0	0	0
Isoleucine	Patients	44	0	0	0
	Controls	14	0	0	0
Leucine	Patients	12	32	32	0
	Controls	14	0	0	0
Valine	Patients	44	0	0	0
	Controls	14	0	0	0
Phenylalanine	Patients	12	32	32	0
	Controls	14	0	0	0
Tyrosine	Patients	44	0	0	0
	Controls	14	0	0	0
Acetate	Patients	10	34	34	0
	Controls	14	0	0	0
Acetoacetate	Patients	12	32	32	0
	Controls	14	0	0	0
3-hydroxybutyrate	Patients	44	0	0	0
	Controls	14	0	0	0
Creatinine	Patients	29	15	15	0
	Controls	14	0	0	0
Albumin	Patients	44	0	0	0
	Controls	14	0	0	0

Glycoprotein acetyls	Patients	44	0	0	0
	Controls	14	0	0	0
